# Laparoscopic advanced intraoperative restaging for radiographic non-metastasis pancreatic cancer: Protocol for a single-center, cross-sectional and follow-up study

**DOI:** 10.1097/MD.0000000000022090

**Published:** 2020-09-04

**Authors:** Zhi Zheng, Ang Li, Feng Cao, Fei Li

**Affiliations:** Department of General Surgery, Xuan Wu Hospital, Capital Medical University, Beijing 100053, China.

**Keywords:** intraoperative restaging, laparoscopy, metastasis, pancreatic cancer, pancreatic surgery

## Abstract

**Background:**

Although surgical resection holds promise for curing pancreatic cancer, <20% of patients are suitable; however, early postoperative recurrence is common. Currently, radiographic examination is the primary method to determine whether pancreatic cancer has metastasized and to inform clinical staging before surgery. However, the method has a limited detection rate for micro-metastasis within the abdominal cavity; therefore, patients with advanced pancreatic cancer and existing micro-metastasis may receive unnecessary surgical treatment, delaying the timing of adjuvant chemotherapy and resulting in poor prognosis. Laparoscopic staging might be used as a supplement to detect micro-metastasis in patients with pancreatic cancer; however, there is no consistent standard to guide the use of this procedure. Therefore, it is necessary to conduct a trial to further explore the consistency and short-term and long-term efficacy of an intraoperative staging strategy for patients with radiographic non-metastasis.

**Methods/design:**

This is a single-center cross-sectional and follow-up study. Patients diagnosed with pancreatic cancer without metastasis by radiographic examination and histopathological biopsy, who received intraoperative restaging, will be enrolled. The total sample size required for the trial is approximately 125 patients from May 2020 to December 2022. First, radiographic examination staging will be used. Then, laparoscopic exploration will be performed for patients without definite metastatic lesions. Data collection will include preoperative blood examination, radiographic examination, surgical information, and postoperative recovery. The patients will undergo follow-up every 3 months after surgery until death. The primary endpoint is the metastasis-positive rate via laparoscopic exploration. The secondary endpoints are the consistency, sensitivity, and specificity of the intraoperative restaging strategy and radiographic examination, the incidence of postoperative complications within 30 days, the 6-month relapse-free survival rate, and perioperative indicators (total cost, hospital stay, length of surgery, and intraoperative blood loss).

**Discussion:**

We are conducting the trial to explore the metastasis-positive rate of intraoperative restaging strategy for diagnosing pancreatic cancer micro-metastasis. This new intraoperative restaging strategy would help pancreatic cancer patients with potential micro-metastasis avoid receiving unnecessary resection, allow systemic treatment as early as possible, and improve the prognosis of patients.

## 1 Introduction

Due to the high degree of malignancy of pancreatic cancer, distant metastases may exist before diagnosis, resulting in rapid disease progression. Some studies have reported that 55% of pancreatic cancer patients have distant metastasis at their initial diagnosis, resulting in a worse prognosis.^[1]^ With the development of systemic treatment, the overall prognosis of pancreatic cancer has improved, and the 5-year survival rate is approximately 9.3%. Although surgical resection holds promise for curing early pancreatic cancer, less than 20% of patients are suitable for radical resection. Even after radical surgery, early postoperative recurrence is common, and for most patients, liver metastases are identified in a short time.^[2,3]^ Surgeons have long been concerned about the potential for radical resection, resulting in a series of concepts and criteria associated with resectability, and characterizing tumors as resectable, borderline resectable, and unresectable.^[4–7]^ Although the related definition clearly indicated that distant organ metastasis belonged to the category of unresectable, it did not clarify how to systematically diagnose or exclude distant metastasis. Currently, the commonly used method of clinical diagnosis is to determine whether the tumor is resectable by preoperative radiographic examination, such as abdominal and pelvic enhanced computed tomography (CT) scan and abdominal magnetic resonance imaging (MRI). Considering the dire circumstances of early distant metastasis after resection of pancreatic cancer, micro-metastasis before surgery has been suspected. However, conventional radiographic examination has a low detection rate for micro-metastasis in the abdominal cavity. Therefore, if some patients with potential micro-metastasis received unnecessary surgical treatment, they generally need 6 to 8 weeks before they are able to receive systemic chemotherapy, which is unfavorable for M1 pancreatic cancer and has a survival period of only 3 to 6 months.^[8,9]^ Laparoscopic staging might be used as a supplement to detect micro-metastasis in patients with pancreatic cancer. However, there is no consistent and uniform surgical procedure. Consequently, it is imperative to perform intraoperative restaging of pancreatic cancer to improve therapeutic strategy.

## 2 Methods/design

### 2.1 Study objectives

This study will explore intraoperative restaging of pancreatic cancer. The purpose is to identify patients with pancreatic cancer without definite metastasis by conventional radiographic examination and conduct intraoperative restaging to identify those with potential metastases (M1) to avoid unnecessary resection, carry out systemic treatment as early as possible, and improve the patient prognosis.

### 2.2 Trial design and patient recruitment

**Figure 1:**
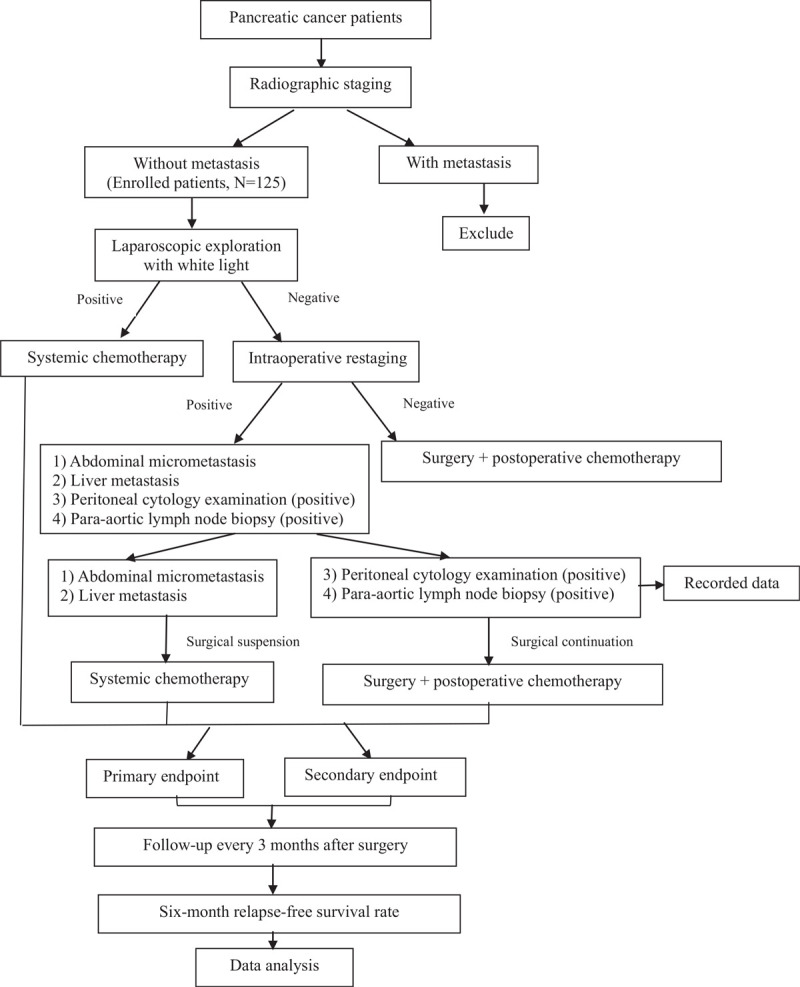
Research process and flow chart.

This study is a single-center, cross-sectional and follow-up study. Enrolled patients will undergo laparoscopic exploration before surgery. The clinical trial was launched in May 2020 and is scheduled to end in May 2023, with enrollment to be completed by December 2022. From May 2020 to December 2022, patients will be selected from Xuan Wu Hospital, Capital Medical University for treatment. All enrolled patients will meet the inclusion criteria (see below). A total of 125 patients are expected to be enrolled in the trial. After completing informed consent forms, patients will receive white light laparoscopic exploration and laparoscopic intraoperative restaging procedures in turn. The detailed research process is described in Figure 1.

### 2.3 Eligibility and inclusion criteria

(1) Patients diagnosed with pancreatic cancer without distant metastasis by traditional radiographic examination, including resectable pancreatic cancer, borderline resectable pancreatic cancer, and locally advanced pancreatic cancer.
(2) Patients aged 18 to 75 years old, regardless of gender.
(3) Patients with Eastern Cooperative Oncology Group (ECOG) performance status ≤2, American Society of Anesthesiologists (ASA) physical status ≤2, and tolerant to radical surgery, including pancreaticoduodenectomy and distal pancreatectomy.
(4) Patients able to sign informed consent on their own or via their legal agent.
(5) Patients without prior history of digestive system tumors, chemotherapy, or radiotherapy.
(6) Patients able to follow the research protocol and follow-up plan.

### 2.4 Exclusion criteria

(1) Patients suffering from uncontrollable diseases, such as unstable angina, myocardial infarction, and cerebrovascular accident that occurred within 6 months.
(2) Patients unwilling to receive surgery.
(3) Patients with confirmed distant metastases, to sites including liver, lung, brain, bone, and non-local lymph nodes.
(4) Patients with history of malignant tumors of other organs within 5 years.
(5) Postoperative histological pathology that confirms non-pancreatic cancer.
(6) Patients unable to receive anesthesia or undergo surgery due to the conditions of other organs.

### 2.5 Elimination criteria

(1) Patients who violate the protocol and receive other anti-tumor treatments during the observation period.
(2) Patients who cannot follow the study treatment protocol.
(3) Incomplete clinical data after enrollment that makes it unsuitable for inclusion in future statistical analysis.

### 2.6 Participating surgeons

Studies have found that surgeon proficiency is significantly related to rates of postoperative complication, mortality, and presence of residual tumor.^[10,11]^ At the same time, there was a study that analyzed surgeons’ first 100 operations and found that a surgeon had completed the surgery learning curve after performing 40 operations and had the ability to handle emergency situations independently during surgery. The length of surgery, blood loss, and incidence of intraoperative and postoperative complications also decreased.^[12]^ Therefore, all surgeons involved in this study will have completed at least 40 pancreaticoduodenectomy or distal pancreatectomy surgeries to ensure the quality of treatment for patients enrolled in the clinical study. We also have several experienced pancreatic surgeons on our research team available to perform surgeries for enrolled patients.

### 2.7 Ethics approval and informed consent

This study has been approved by the Ethics committee of the Xuan Wu Hospital, Capital Medical University. According to the requirements of the ethics committee, clinical research will be conducted only after the enrolled patients have signed the informed consent forms. The design of this study is consistent with the principles of the Declaration of Helsinki. All data will be recorded and analyzed anonymously to protect patient privacy. The trial was registered in May 2020 on the Chinese Clinical Trial Registry website (http://www.chictr.org.cn/index.aspx). The registration number is ChiCTR2000032628. Documentation of informed consent is required from all enrolled patients before the study initiation. Participating patients will be informed of the purpose and significance of the study, the benefits and possible risks of participation, and the confidentiality of the study. Enrolled patients will have the opportunity to ask questions and receive answers.

### 2.8 Interventions

#### 2.8.1 Intraoperative restaging via laparoscopic exploration

In this study, the intraoperative restaging strategy will be implemented for all enrolled patients. Before laparoscopic exploration, traditional radiographic examination staging will be used. Pancreatic cancer patients will have undergone abdominal MRI, abdominal and pelvic enhanced CT scan, and assessment for tumor markers before surgery to determine whether they had intra-abdominal micro-metastasis, liver metastases, peritoneal effusion, or abnormal non-localized lymph node enlargement. For patients without definite metastatic lesions, laparoscopic exploration will be performed.

Surgery in the supine position will be used in all cases, and laparoscopic exploration will be performed after successful general anesthesia and tracheal intubation. A 10 mm Trocar will be inserted through a transverse incision 1.5 cm below the umbilicus to establish the pneumoperitoneum and maintain pressure at the 12 to 15 mmHg level. The intraperitoneal lens will be inserted, the 5 mm trocar will be placed in the midline of the right clavicle under direct view, and the surgical forceps will be inserted. First, we will perform laparoscopic exploration with white light (detailed surgical procedures were described in the first part of the laparoscopic restaging strategy). Then, if white light laparoscopy does not detect abdominal micro-metastasis, we will perform laparoscopic restaging prior to the pancreaticoduodenectomy or distal pancreatectomy to further determine whether the tumor had spread via micro-metastasis.

This laparoscopic restaging strategy includes 4 main aspects: First, fluorescent laparoscopy will be used to explore the abdominal cavity to determine whether there is a micrometastatic lesion. The order of exploration is left and right subdiaphragm→liver and spleen→abdominal wall peritoneum→pelvis→omentum, small intestine, and mesentery→transverse colon mesentery→stomach→small omentum sac→duodenum→pancreas. During surgical exploration, special attention will be paid to micro-metastasis on the surface of the right posterior lobe of the liver, the surface of the proximal jejunum, the small omentum sac, and the retroperitoneal area around the duodenum. Suspicious metastatic nodules will be taken for pathological examination, if necessary. Next will be examination of peritoneal cytology. The irrigating tube will be placed in the 5 mm trocar, and the peritoneum and mesentery of the left and right subphrenic, abdominal pelvic cavity will be irrigated with 500 ml sterile saline. The patient's head will be lifted and their feet lowered to collect the abdominal cavity lavage fluid from the Douglas cavity, the liver, and the spleen fossa to determine whether cancer cells have been shed into the abdominal cavity. Third will be laparoscopic liver ultrasound. In addition to routine inspections during surgery, near-infrared (NIR) imaging and intraoperative ultrasound will be used to examine the liver.^[13]^ After detection, an ultrasound contrast agent will be injected; the recommended contrast agent that allows assessment of Kupffer cell phase will be used. After the completion of the No. 16 lymph node biopsy, the intraoperative ultrasound will be performed again to detect liver metastasis. Finally, abdominal aortic lymph node (No. 16) biopsy will be performed. Pancreatic head cancer will be biopsied through the Kocher incision for vena cava interaortic lymph nodes (No. 16a2 and No. 16b1). For pancreatic body and tail cancer, except for the above areas, the para-aortic lymph nodes will be explored through the Treitz ligament.^[14]^ If no clear micro-metastasis is visible, the surgeon may perform radical surgery. Otherwise, surgeons will complete the operation and commence postoperative chemotherapy as quickly as possible. Every patient will have surgery performed by an experienced surgeon. Positive peritoneal cytology and abdominal aortic lymph node (No. 16) biopsy are not indications of surgical suspension. However, detailed data will be maintained in medical records for future analysis.

#### 2.8.2 Perioperative treatment for enrolled patients

(1) For patients with clearly identified abdominal organ micro-metastasis by intraoperative restaging via laparoscopic exploration, there will be no need for radical surgery. Patients will need only short-term total parenteral nutrition support after surgery. Upon intestinal function recovery, patients will be given a liquid or semi-liquid diet. Adjuvant chemotherapy will be started 2 to 3 weeks after surgery.
(2) For patients who received radical surgery, clinicians will give symptom-based treatment, such as antibiotics, proton pump inhibitors, analgesics, octreotide, total parenteral nutrition support, and blood products according to the patient's recovery. Routine postoperative blood tests, biochemistry tests, blood amylase, and abdominal drainage fluid amylase will be reviewed regularly to monitor for anastomotic leakage, delayed bleeding, and pancreatic leakage. There will be regular abdominal ultrasound examination to monitor for peritoneal effusion. If necessary, drainage by abdominal puncture will be performed. After return to a normal diet, patients will receive adjuvant chemotherapy 6 to 8 weeks after surgery.

### 2.9 Primary endpoint

The primary endpoint of the study will be the metastasis-positive rate of laparoscopic exploration. The positive rate of laparoscopic exploration is defined as the patients with pancreatic cancer with peritoneal micro-metastasis, liver metastases, para-aortic lymph node metastases, and peritoneal cytology with abnormal cells or tumor cells identified during laparoscopic exploration surgery. A patient meeting any one or several of the above indicators would be considered positive via laparoscopic exploration of pancreatic cancer. However, positive peritoneal cytology examination and abdominal aortic lymph node (No. 16) biopsy are not indications of surgical suspension.

### 2.10 Secondary endpoint

The secondary endpoints are:

(1) Consistency, sensitivity, and specificity of the intraoperative restaging strategy and traditional radiographic examination for the diagnosis of pancreatic cancer micro-metastasis.
(2) Total cost of hospitalization.
(3) Total duration of hospitalization.
(4) Duration of surgery, including laparoscopic exploration and total estimated intraoperative blood loss.
(5) The incidence of postoperative complications within 30 days, including anastomotic fistula, pancreatic fistula, intestinal obstruction, delayed bleeding, and incision-related complications. According to the Clavien-Dindo classification, the postoperative complications classified as higher than Grade II will be regarded as clinically significant (Table 1).^[15]^
(6) Six-month relapse-free survival rate (RFS) defined as the time interval from the date of surgery until detection of tumor recurrence within the 6-month period after surgery.

**Table 1:**
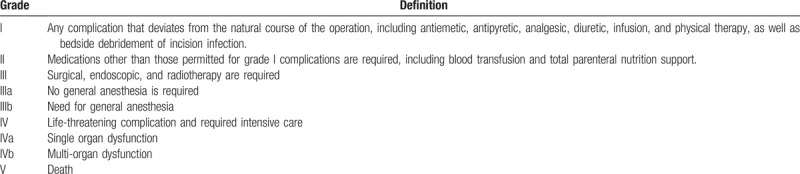
Clavien-Dindo classification.

### 2.11 Follow-up

Post-operative follow-up will be carried out by a dedicated staff member. The patients will be assessed via follow-up every 3 months after surgery. After discharge, follow-up will occur via outpatient visit, telephone, or mail. During the follow-up period, the patients will receive physical examination, laboratory tests, and chest and abdominal CT scan or abdominal ultrasound assessment. The laboratory tests will include routine blood tests, blood biochemistry, and assessment for tumor markers such as CA19-9, CA125, AFP, CEA, and CA724. If tumor recurrence or distant metastasis is identified after surgery, further detailed evaluation will be required, and will include abdominal MRI or positron emission tomography (PET)/CT to determine whether surgical resection is feasible. The patients will be followed until death.

### 2.12 Adverse events

All serious adverse events (SAEs) occurring between the signing of the informed consent and the end of the trial need to be recorded. SAEs are defined as any injury related to or not related to the expected outcome of the operation. The trial includes an independent data monitoring committee that will review the ongoing safety data in an unblinded manner in accordance with the Standard Operation Procedures for Clinical Trials, Japan Medical Association. All the patients will be received the best treatments for curing complications.

### 2.13 Data collection

A standard and uniform case report form (CRF) has been designed and the electronic data capture (EDC) system has been established. All data will be required to be collected via the CRF. The clinical research coordinator (CRC) will enter the data into the EDC system in a timely manner. Every month, clinical research associates will monitor the electronic database to guarantee the quality of data.

Data will be collected via the CRF as follows: (1) Preoperative blood examination, including the results of routine blood tests, biochemical investigations, and tumor markers, such as CA19-9, CA125, AFP, CEA, and CA724. (2) Radiographic examination, including which enrolled patients require abdominal enhanced CT scan and MRI, and whether PET/CT examination is feasible, if necessary, and results of radiographic assessment. (3) Surgical information, including the date and time of surgery, blood loss, and surgical methods. (4) Postoperative recovery and follow-up outcomes, including total cost of hospitalization, length of hospital stay, postoperative complications, and death (Table 2).

**Table 2:**
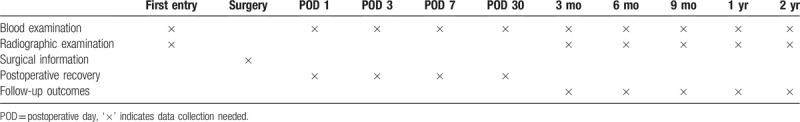
Checklist for enrolled pancreatic cancer patients.

During the study, all personal data, such as name and sex, will be replaced with statistical codes or numbers and will be kept strictly confidential. All clinical data will be analyzed anonymously through the CRF and EDC system to protect the privacy of the patients. We will transfer the CRFs to the EDC (https://edc-cloud.medsci.cn/#/login), which will be stored in a hard disk and cloud environment. Detailed results will be openly shared after study completion.

### 2.14 Sample size

The necessary sample size was calculated using PASS 11.0 (NCSS Statistical and Data Analysis, USA) software. It is estimated based on the preliminary research results from our center. This study is a cross-sectional and follow-up study. The patients will receive white light laparoscopic exploration and intraoperative restaging via laparoscopic exploration in turn. According to the single group design, the rate of micro-metastasis detection via laparoscopic exploration with white light laparoscopy is 10%, while the detection rate via the intraoperative restaging strategy is 25%, with 8% allowable error. Using a one-sided test, the α value was equal to 0.05. The estimated total sample size required for the trial is at least 113 patients. The withdrawal rate is assumed to be 10% during follow-up. Therefore, a total sample size of 125 patients for the study will be required.

### 2.15 Statistical analysis

The SPSS 21.0 (IBM Corp., Armonk, NY, USA) statistical software will be used for statistical analysis. The measurement data will be in accordance with the normal or approximate normal distribution, and the comparison between multiple groups will be assessed via univariate analysis of variance. The non-normal distribution data will be expressed as median (interquartile range) and compared using the Kruskal-Wallis *H* test. The count data will be expressed as frequency and percentages and will be compared using the *χ*^2^ test, corrective *χ*^2^ test, or Fisher's exact test. Ranked data will be expressed as frequency and percentages and will be compared using the rank sum test. Sensitivity and specificity will be calculated to evaluate the diagnostic performance. The Kaplan-Meier method will be used to draw survival curves, and the survival rates of multiple groups will be compared using the log-rank test. The univariate and multivariate Cox proportional hazards model will be used to evaluate the hazard ratios (HRs) for adverse outcomes. All statistical tests will be one-sided, with a *P* value <.05 considered statistically significant.

### 2.16 Monitoring and quality assurance

The trial has a research supervision committee, and the members of the committee have a clear division of labor and cooperation. The supervision committee consists of data managers, data inspectors, and methodological teams. Each part of the clinical trial has a standard operation procedure (SOP) to ensure the homogeneity of the research. Meanwhile, there are specialists engaged in data collection, data entry, data cleaning, and patient follow-up.

According to the research data and observation indicators, the CRF table was constructed by the researchers and submitted to the ethics committee for review after completion. After passing the review, the data manager will create a CRF table of individual patients based on the information provided by the researchers. The clinical investigators will designate a dedicated data entry officer to enter the research data on the CRF table in a timely and accurate manner, and the data inspectors will confirm that the CRF table is complete and correct. After the study data are verified by the data inspector, the data manager shall archive the data until the data collection for the last enrolled patient is complete. After all the data are archived, researchers will submit data to the methodology team for statistical analysis.

### 2.17 Patient and public involvement

Patients and the public were/are not involved in the design, recruitment, or conduct of the trial.

### 2.18 Dissemination plans

We will publish the results of the trial in professional peer-reviewed journals after all data have been collected and analyzed.

### 2.19 Trial status

Version 1.0 of this trial was approved in April 2020. The trial was registered in May 2020 on the Chinese Clinical Trial Registry website (http://www.chictr.org.cn/index.aspx). Although we do not enroll the pancreatic cancer patients, we have developed related SOP, CRF, and EDC systems. In May 2020, our trial began enrollment of patients for research on schedule, and the recruitment is expected to be completed in December 2022. We plan to publish our data in May 2023.

## 3 Discussion

Because of the high degree of malignancy of pancreatic cancer, the 5-year survival rate of some patients is less than 10%, even after radical surgery.^[2]^ Early postoperative recurrence is associated with poor overall prognosis in patients with pancreatic cancer. A study reported recurrence among 692 patients with pancreatic cancer who underwent resection, at the median follow-up of 25.3 months. A total of 531 (76.7%) patients had relapsed, with a median relapse time of 11.7 months. Among all patients with relapse, liver metastasis was the most common (134 patients, 25.2%), and the median recurrence time was only 6.9 months in this group.^[16]^ Other research summarized the time distribution characteristics of postoperative recurrence of pancreatic cancer. Among 957 patients with median follow-up of 24.2 months, 753 (78.7%) had recurrence. There were 85 cases (11.3%), 182 cases (24.1%), 388 cases (51.5%), and 526 cases (69.9%) at 3, 6, 12, and 18 months, respectively. The study confirmed that early recurrence is significantly associated with poor prognosis among pancreatic cancer patients.^[3]^ Another study found that lymph node status was closely related to postoperative recurrence time. The patients with lymph node metastasis (N1) have a shorter relapse-free survival time (RFS), and neoadjuvant therapy may prolong RFS.^[17]^ Therefore, accurately determining whether micro-metastasis has occurred in patients with pancreatic cancer before performing tumor resection and formulating appropriate treatment plans is still fertile ground for research. Meanwhile, intraoperative restaging strategy models this treatment concept. Complete intraoperative restaging should include, at minimum, the following aspects: (1) exclusion of abdominal micro-metastasis; (2) peritoneal cytology examination; (3) exclusion of liver metastasis; (4) para-aortic lymph node biopsy.^[18–21]^

In recent years, minimally invasive surgery has developed rapidly, and laparoscopic technology has been used in all aspects of pancreatic surgery. Based on current research, although the laparoscopic learning curve is longer, laparoscopic pancreatectomy is one of the safest and most feasible treatment methods.^[22]^ The strategy of intraoperative restaging compliments the clinical work already carried out and does not increase the difficulty of surgery. From our prior experience, there is only a slight increase in the operation time, and intraoperative restaging does not affect the safety of surgery. Reasonable arrangement of intraoperative restaged surgical procedures helps shorten the length of surgery. However, there is still no evidence indicating whether this intraoperative restaging strategy can effectively improve the detection of micro-metastasis that cannot be detected by traditional radiographic examination. Consequently, it is necessary for us to carry out this clinical trial to clarify the intraoperative restaging strategy and the surgical indications of pancreatic cancer patients to inform improved treatment plans. It is expected that patients with potential micro-metastasis can avoid unnecessary radical surgery to some extent, allowing systemic treatment as early as possible and prolonging the prognosis of pancreatic cancer.

This study has certain limitations: (1) As many patients with pancreatic cancer in the early stages are asymptomatic, most patients are already in advanced stages when symptoms appear. The number of patients who can receive radical surgery is small, resulting in a small sample size of patients with pancreatic cancer who meet the inclusion criteria for our trial. Therefore, there is a certain degree of data bias which may not fully represent the overall disease status and long-term prognosis of pancreatic cancer patients. (2) This study is a single-center, cross-sectional and follow-up study; therefore, the quality of evidence will be lower than that of a large-sample, multi-center, randomized, controlled study. However, a successful trial will provide important data upon which to build via iterations of the research protocol in future stages. Trial expansion will occur through inclusion of additional research centers and increased patient enrollment as we strive for more precise treatment for pancreatic cancer and improved long-term prognosis for pancreatic cancer patients.

## Acknowledgments

We would like to thank Editage (www.editage.cn) for English language editing.

## Author contributions

Fei Li is the principal researcher who carried out all phases of trial design. Zhi Zheng participated in collecting data and signed informed consent documents. Ang Li performed the statistical analysis and participated in its design. Feng Cao and Ang Li are responsible for formulating the Standard Operation Procedure for surgery. Zhi Zheng helped draft the manuscript. Ang Li and Feng Cao revised the manuscript. All authors read and approved the final manuscript.
